# Molecular characteristics of *Mycobacterium tuberculosis* drug-resistant isolates from HIV- and HIV+ tuberculosis patients in Russia

**DOI:** 10.1186/s12866-022-02553-7

**Published:** 2022-05-19

**Authors:** Anna E. Panova, Anatoliy S. Vinokurov, Anastasiya A. Shemetova, Irina A. Burmistrova, Marina V. Shulgina, Anastasiya G. Samoilova, Irina A. Vasilyeva, Diana V. Vakhrusheva, Tatiana V. Umpeleva, Nataliya I. Eremeeva, Leonid S. Lavrenchuk, Lyudmila A. Golubeva, Tatiana I. Danilova, Tatiana B. Vasilyeva, Vera A. Ugol’kova, Nataliya V. Sosova, Marina V. Lekhlyaider, Irina A. Gorshkova, Tatiana A. Romanova

**Affiliations:** 1 National Medical Research Center of Phthisiopulmonology and Infectious Diseases, Ministry of Public Heath of the Russian Federation (NMRC PhID), Moscow, Russian Federation; 2 Ural Research Institute of Phthisiopulmonology -Branch of NMRC PhID, Ekaterinburg, Russian Federation; 3 Regional TB dispensary of Leningradskaya oblast, Saint Petersburg, Russian Federation; 4 Regional TB dispensary of Stavropolskiy kray, Stavropol, Russian Federation; 5 Regional TB dispensary of Chelyabinskaya oblast, Chelyabinsk, Russian Federation; 6 Regional TB dispensary of Kemerovskaya oblast, Kemerovo, Russian Federation

**Keywords:** *Mycobacterium tuberculosis*, Genotypes, Drug-resistance conferring mutations, Beijing

## Abstract

**Background:**

High burden of drug-resistant (DR) tuberculosis (TB) is a significant threat to national TB control programs all over the world and in the Russian Federation. Different *Mycobacterium tuberculosis* (MTB) genotypes are hypothesized to have specific characteristics affecting TB control programs. For example, Beijing strains are supposed to have higher mutation rates compared to strains of other genotypes and subsequently higher capability to develop drug-resistance.

**Results:**

Clinical MTB isolates from HIV- and HIV+ patients from four regions of Russia were analyzed for genotypes and mutations conferring resistance to Isoniazid, Rifampicin, Ethambutol, aminoglycosides, and fluoroquinolones. Analysis of genotypes and polymorphism of genomic loci according to the HIV status of the patients – sources of MTB isolates were performed. Studied MTB isolates from HIV- TB patients belonged to 15 genotypes and from HIV + TB patients – to 6 genotypes. Beijing clinical isolates dominated in HIV- (64,7%) and HIV+ (74,4%) groups. Other isolates were of LAM (including LAM1 and LAM9), Ural, and 4 minor groups of genotypes (including 5 subclones T). The spectrum of genotypes in the HIV- group was broader than in the HIV+ group. PR of B0/W148 Beijing was significantly lower than of other Beijing genotypes in susceptible and MDR-XDR isolates. Rates of isolates belonging to non-Beijing genotypes were higher than Beijing in susceptible isolates from HIV- patients.

**Conclusions:**

Beijing genotype isolates prevailed in clinical isolates of all drug susceptibility profiles both from HIV- and HIV+ patients, although B0/W148 Beijing genotype did not dominate in this study. Genome loci and mutations polymorphisms were more pronounced in clinical isolates from HIV- patients, than from HIV+.

## Background

High burden of drug resistant (DR) tuberculosis (TB) is a significant threat to national TB control programs all over the world [1, 2]. The Russian Federation (RF) is in the group of countries with high level emergence of multidrug resistant (MDR) TB, with estimated proportion of MDR TB cases in 2019 amounting 35% of new cases and 71% of previously treated cases. Rates of HIV positive (HIV+) patients with TB are also high [1].

Geographic distribution of *Mycobacterium tuberculosis* (Mtb) genotypes is of unrelenting interest of the researchers in many countries. Data on genotyping as specific characteristics of MTB strains gives a new impulse to developing molecular epidemiology, and induced discussions on molecular peculiarities of Mtb, causing disease in a particular region of the world. Results of numerous researches on Mtb genotyping using spoligotyping, MIRU-VNTR typing and whole genome sequencing are accumulate in electronic databases, for example in Pasteur Institute database [3]. Most of these investigations are aimed at phylogeny and evolution of MTB.

Studies of Mtb genotypes circulating at the territory of the Russian Federation have been going on for decades. The prevailing genotype is Beijing. Beijing genotype belongs to East-Asian lineage (lineage 2) and widely spreads all over the world [4, 5]. Beijing strains are abundant in East Asia and in the former Soviet Union republics. They are frequently isolated from TB patients in Russia and among immigrants from the former Soviet Union. Strains belonging to Beijing subclone BO/W148 are isolated from TB patients all over the territory of the Russian Federation [5, 6]. Other Mtb genotypes isolated at the territory of the Russian Federation less frequently than Beijing strains are genotypes of Euro-American family, such as LAM, Ural, Haarlem, S, T and X-types and others [7, 8].

Different MTB genotypes are hypothesized to have specific characteristics effecting TB control programs. Beijing strains are supposed to have higher mutation rates compared to strains of other genotypes and subsequently higher capability to develop drug-resistance [5, 9–11]. Beijing genotype is also associated with unfavorable outcomes of TB treatment [12]. Other researchers have demonstrated that Beijing strains had not developed resistance to rifampin in elevated rate and there were no significant differences in occurrence of mutations between Beijing and non-Beijing clinical isolates (ClIs) [13–15].

Currently the best method for revealing Mtb genotyping and molecular DR profile is whole genome sequencing [16, 17]. However, the use of this method is limited by high level technology laboratories. Use of commercial genotyping test-systems made it possible for regional clinical laboratories to be included in the pool of centers performing molecular epidemiological surveys and to introduce molecular characteristics of an etiological agent to routine clinical practice. These researches provide more data on the mechanisms of DR and mutations conferring DR phenotype of Mtb to develop and update tests for DR TB diagnosis and personalized chemotherapy regimens.

In 2018–2019 the National Medical Research Center of Phthisiopulmonology and Infectious Diseases (NMRC PhID) and its’ branch the Ural Research Institute of Phthisiopulmonology (URIPh) had collected Mtb ClIs from different regions of Russia for their drug sensitivity testing (DST) reanalysis as a part of the regional External Quality Control program. Subsequently molecular characteristics of the ClIs, including their genotyping and testing for mutations associated with Mtb DR were studied. ClIs were attributed according to HIV status of TB patients. In this research we had evaluated rates of different genotypes according to clinical Mtb ClIs’ DR profiles of loci and types of mutations conferring DR. The aim of this study was to analyze polymorphisms of different DNA loci associated with Mtb DR according to the ClIs’ genotypes and HIV status of patients, who provided material for ClIs.

## Results

### DR profiles and genotypes in Mtb ClIs from HIV- and HIV+ TB patients

Mtb ClIs from HIV- TB patients belonged to 15 genotypes (Table 1). 280 ClIs (PR 64,7%, CI95% = 4,5%) were Beijing, including 82 B0/W148 Beijing (PR 19,0%, CI95% = 3,7%), 76 ClIs (PR 17,7%, CI95% = 3,6%) belonged to LAM genotype (including LAM1 and LAM9–1 isolate each), 33 ClIs (PR 7,7%, CI95% = 2,5%) to Ural genotype. 42 ClIs belonged to 10 subfamilies (minor genotypes), 2 ClIs did not belong to genotypes, identified by the test-system used.

**Table 1:** Genotypes of Mtb clinical ClIs

	ClIs from HIV- patients			ClIs from HIV+ patients		
Genotypes	No	PR, %	CI, 95%^a^	No	PR, %	CI, 95%
B0 / W148 Beijing	82	18,9	3,7	26	20,2	6,9
Other Beijing	198	45,7	4,7	70	54,3	8,6
Ural	35	8,1	2,6	19	14,7	6,1
LAM	74	17,1	3,5	10	7,8	4,6
LAM 1	1	0,2	0,5	0	0	0
LAM 9	1	0,2	0,5	0	0	0
EAL	2	0,5	0,6	1	0,8	2,0
T 1	14	3,2	1,7	3	2,3	3,0
T 1 RUS 2	5	1,2	1,0	0	0	0
T 2	2	0,5	0,6	0	0	0
T 3	4	0,9	0,9	0	0	0
T 4	2	0,5	0,6	0	0	0
X 1	5	1,2	1,0	0	0	0
Haarlem	6	1,4	1,1	0	0	0
Not identified	2	0,5	0,6	0	0	0
TOTAL	433	100		129	100	

^a^+/= CI – 95% confidence interval for Prevalence value (PR)

In the group of ClIs from HIV + TB patients’ number of Beijing ClIs was 96 (PR 74, 4%, CI95% = 7, 5%) including 26 B0/W148 Beijing ClIs (PR 20, 2%, CI95% = 6, 9%). LAM genotype ClIs from HIV+ patients PR was 7, 8%, CI95% = 4, 3%. PR for Ural genotype ClIs was 14, 7%, CI95% = 6, 1%. Number of minor genotypes was only 3: EAL, and T1. No ClIs from L1 and L9 genotypes were identified.

PRs of Beijing and Ural ClIs from HIV+ patients were significantly higher than from HIV- patients (*p* = 0, 04 for both). PR for ClIs of LAM genotype was significantly higher in the group from HIV- patients, than in the group of ClIs from HIV+ patients (*p* = 0,008).

Beijing ClIs (including B0/W184 Beijing) were detected in susceptible and DR ClIs from HIV- patients (Table 2). The Beijing (total) fraction in susceptible ClIs (53 of 142 ClIs, PR = 37, 3%, CI95% = 8, 0%) was smaller than in MDR-pre-XDR- XDR (MDR-XDR) ClIs (160 of 199 ClIs PR = 86, 4%, CI95% = 4, 8%) (*p* < < 0, 0001). Differences in rates of B0/W148 Beijing and other Beijing genotypes in susceptible and MDR-XDR ClIs were also statistically significant: number of B0W148 Beijing were 0 of 142 susceptible ClIs and 58 of 199 (PR = 29,1%, CI95% = 6, 6%) in MDR-XDR group (*p* < < 0, 0001), number of other Beijing ClIs – 53 (PR = 37, 3%, CI95% = 8, 0%) and 110 (PR = 55, 3%,, CI95% = 6, 9) respectively, (*p* = 0,0014) (Table 2).

**Table 2:** Number of MTB ClIs belonging to genotype subfamilies and their drug resistance profiles

Drug susceptibility profile	Total No	BEIJING			LAM + LAM1 +LAM2	Ural	Minor genotypes^a^
		Total	B0/W148	Other^b^			
No of ClIs from HIV- patients							
Susceptible	142	53	0	53	37	18	35
Monoresistant	53	32	10	22	7	9	6
Polyresistant	39	35	14	21	9	4	3
MDR	85	69	24	45	15	1	0
preXDR	62	51	25	26	3	1	0
XDR	52	40	9	31	5	0	0
TOTAL	433	280	82	198	76	33	44
No of ClIs from HIV+ patients							
Susceptible	58	15	0	15	6	15	4
Monoresistant	9	7	0	7	2	5	0
Polyresistant	1	6	0	6	2	0	0
MDR	26	40	12	28	0	0	0
preXDR	25	39	8	9	0	0	0
XDR	10	8	6	4	0	0	0
TOTAL	129	95	26	69	10	20	4

^a^Minor genotypes are for HIV-: EAL, Н3, H 4, T 1, T 1 RUS 2, T 2, T 3, T 4, X 1, Haarlem; and for HIV+: EAL, H 4, T 1

^b^ClIs of Beijing genotype other than B0/W148

Rates of non-Beijing genotypes ClIs were higher in susceptible ClIs from HIV- patients. Differences in rates of LAM (including LAM1 and LAM9), URAL and minor genotypes in susceptible and MDR-XDR ClIs were statistically significant: LAM ClIs were 37 (PR = 26, 1%, CI95% = 7, 2%) in susceptible group and 23 (PR = 11, 6%,, CI95% = 4, 4%) in MDR-XDR group (*p* = 0,0014), URAL ClIs – 18 (PR = 12,7%, CI95% = 5, 5%) and 2 ClIs PR = 1, 0%, CI95% = 1, 4%) (*p* = 0,019) and minor genotypes’ ClIs – 35 (PR = 24,6%, CI95% = 7,1%) and 2 (PR = 1, 0%, CI95% = 1, 4%) (*p* < < 0,0001), correspondingly (Table 2).

For ClIs from HIV+ patients statistically significant differences for susceptible (58 ClIs) and MDR-XDR fractions (61 ClIs) were revealed for Beijing (total) fraction: rates were 37 ClIs of 58, (PR = 63, 7%, CI95% = 12, 4%) and 54 ClIs of 61, (PR = 88, 5%, CI95% = 8, 0%) (*p* = 0, 00002), correspondingly. Differences in rates of B0/W148 Beijing and other Beijing genotypes in susceptible and MDR-XDR ClIs were also statistically significant: B0W148 Beijing ClIs were 0 in susceptible group and 26 (PR = 42, 6%, CI95% = 12, 4%) in MDR-XDR group (*p* < < 0, 0001) and PRs of other Beijing ClIs in the groups of susceptible and MDR-XDR ClIs were 37 (PR = 63, 7%, CI95% = 12, 4%) and 28 (PR = 45, 9%, CI95% = 1, 5%) (*p* = 0, 06), correspondingly.

Rate of ClIs from HIV+ patients belonging to Ural genotype was higher in susceptible ClIs compared to MDR -XDR group: 12 (PR = 20, 7%, CI95% = 10, 4%) and 4 (PR = 6, 6%, CI95% = 6,2%), correspondingly., *(p* = 0,03). Differences in rates of LAM and minor genotypes in susceptible and MDR-XDR ClIs were also statistically insignificant: of LAM genotype were 5 (PR = 8, 6%, CI95% = 7, 2%) and (PR = 3, 3%, CI95% = 4, 5%), correspondingly, *p* = 0, 26, and of minor genotypes were 4 susceptible (PR = 6,6%, CI95% = 6, 2%)) and 1 MDR-XDR (PR = 1, 6%, CI95% = 1, 6%) ClIs б *p* = 0, 20 correspondingly (Table 2).

### Resistance to INH

Total number of INH resistant ClIs was 233 from HIV- and 79 from HIV+ patients. All ClIs with mutations in katG and inhA genes, both from HIV- and HIV- patients were resistant to INH according to DST. Mutations in both genes were detected in 21 (PR = 9,0%, CI95% = 3,7%) ClIs from HIV- patients. All ClIs with double mutations were of Beijing genotype (other Beijing), four were monoresistant to INH, 12 – MDR and five – XDR. In all cases mutation were katG315Ser315Thr, inhA15Thr15Ala. No ClIs with double mutations were detected in the group from HIV+ patients. In analysis presented below each of these mutations were considered as mutations in separate ClIs with total number of evaluated ClIs from HIV- patients – 254, and from HIV+ patients – 79 (Table 3).

**Table 3:** Loci and types of mutations conferring INH resistance in MTB clinical isolates belonging to different genotypes

Loci and types of mutations	Number of MTB isolates with mutations conferring INH resistance from HIV - patients						Number of MTB isolates with mutations conferring INH resistance from HIV+ patients				
	BEIJING			LAM	Ural, T1	TOTAL	BEIJING			LAM, Ural	TOTAL
	TOTAL Beijing	B0 / W148 Beijing	Other Beijing				TOTAL Beijing	B0 / W148 Beijing	Other Beijing		
katG 315-Ser315Thr	**166**	**64**	**102**	**27**	**7**	**200**	**73**	**28**	**45**	**0**	**73**
katG 315- Ser315Arg	2	0	2	0	0	**2**	1	0	1	0	**1**
katG 315-Ser315Gly	2	0	2	0	0	**2**	0	0	0	0	**0**
katG 315 Ser315Ile; Ser315Asn	0	0	0	0	0	**0**	0	0	0	0	**0**
katG 328-Trp328Cys	2	0	2	0	0	**2**	0	0	0	0	**0**
katG 328 Trp328Gly; Trp328Leu	0	0	0	0	0	**0**	0	0	0	0	**0**
katG 335-Iley335Val	5	0	5	0	0	**5**	0	0	0	0	**0**
**Total katG**	**176**	**64**	**113**	**27**	**7**	**211**	**74**	**28**	**46**	**0**	**74**
inhA t8a	8	0	8	0	0	**8**	0	0	0	0	**0**
inhA t8g	0	0	0	0	0	**0**	0	0	0	0	**0**
inhA t15a	**23**	**3**	**20**	**11**	**1**	**35**	2	1	1	3	**5**
inhA a16g	0	0	0	0	0	**0**	0	0	0	0	**0**
inhA t24a	0	0	0	0	0	**0**	0	0	0	0	**0**
**Total INH A**	**31**	**3**	**28**	**11**	**1**	**43**	**2**	**1**	**1**	**3**	**5**

0 :- loci and types of mutations that were tested but not revealed

In one isolate a mutation often associated with INH resistance - ahpC12 (Cys12Thr) as a sole mutation was detected, however the isolate was susceptible to INH in DST.

Beijing genotypes prevailed in ClIs with katG from HIV- patients: Beijing genotypes – 176 ClIs of 211 (PR = 83,4%, CI95% = 5,0%), LAM – 27 ClIs (PR = 12,8%, CI95% = 4,5%) and other genotypes (Ural and T1) – 7 ClIs (PR 3,3%, CI95% = 2,4%). Rate of B0/W148 Beijing (64 ClIs of 211) PR = 30,3%, CI95% = 6,2%, was significantly lower than others Beijing (113 ClIs of 211), (PR = 53,6%, CI95% = 6,7%) (*p* = 0,00001). Similarly, Beijing genotype was the most frequently detected in ClIs with inhA mutations – 31 of all 43 (PR = 72,1% CI95% = 13,4%), LAM – 11 (PR = 25,6%, CI95% = 13,0%) and Ural – 1 (PR 2,3%, CI95% = 4,5%) ClIs. Rate of B0/W148 Beijing (3 ClIs of 43) PR = 7,0%, CI95% = 7,6%, was significantly lower than others Beijing ClIs (28 ClIs of 43), (PR = 65,1%, CI95% = 14,2%) (*p* < < 0,0001).

In HIV+ group of ClIs with katG mutations only Beijing genotypes were detected (74 ClIs). 28 ClIs were of BO/W148 (PR = 37,8%=/−CI95% = 11,1%), 46 – were of other Beijing (PR = 62,2%=/−CI95% = 11,1%). Two ClIs with inhA mutations belonged to Beijing genotype (one to B0/W148 and one to other Beijing subclones), two to LAM and one to Ural genotypes.

In HIV- group of 254 ClIs 211 harbored mutations in katG gene (PR = 83,1%, CI95% = 4,6%), and 43 in inhA gene (PR-16,9%, CI95% = 4,6%). INH mutations were detected in four loci of katG gene: three types of mutations at katG315- substitution of Serine by Threonine, Arginine and Glycine, at katG328 - Tryptophan by Cysteine and at katG335-Isoleucine by Valine (see Table 3).

Prevailing type of mutation in katG was Ser315Thr: 200 of 211 ClIs (PR = 94,8%, CI95% = 3,0%) resistant to INH. It was associated with Beijing: 166 ClIs with Ser315Thr mutations were detected in 176 Beijing ClIs (PR = 94,3%, CI95% = 3,4%), 27 in LAM ClIs (PR = 15,3%, CI95% = 5,3%) and 7 in other genotypes’ ClIs (PR = 4,0%, CI95% = 2,9%). Differences between Beijing and other genotypic groups were statistically significant (*p* < < 0,0001 for both groups). Rate of B0/W148 Beijing ClIs (64 of all 166 Beijing ClIs, PR = 38,6%, CI95% = 7,4%) harboring this mutation was significantly lower than of other Beijing ClIs (102, PR = 61,4%, CI95% = 7,4%) (*p* = 0,00001).

Two types of mutations were detected in inhA gene – t8a and t15a. Mutations t8g, a16g, t24g were not detected. Among inhA mutations 35 of all 43 inhA harboring ClIs were t15a (PR = 81,4%, CI95% = 11,6%). It was associated with Beijing genotype: 23 of 35 ClIs with this mutation (PR = 65,7%, CI95% = 15,7%) belonged to Beijing genotype. Rate of B0/W148 Beijing ClIs harboring this mutation (3 ClIs, PR = 8,6%, CI95% = 9,3%) was significantly lower than of other Beijing ClIs (20 ClIs PR = 57,1%, CI95% = 16,4%) (*p* = 0,00005).

In HIV+ group of ClIs only one locus in katG gene with two types of mutations was detected: katG315- substitution of Serine by Threonine and Arginine. Ser315Thr mutation was detected in73 of 74 ClIs (PR = 98,7%, CI95% = 2,6%). Rate of ClIs with this mutation was lower among B0/W148 Beijing than in other Beijing ClIs – 28 and 45 of 73, PR = 38,4%, CI95% = 11,2%and 61,6%, CI95% = 11,2%, correspondingly (*p* = 0,0014). In inhA gene only t15a mutations were detected.

### Resistance to RIF

Total number of ClIs from HIV- patients bearing mutations associated with resistance to RIF was 215, and of ClIs from HIV+ patients - 62. Mutations in rpoB gene were only detected (Table 4). All ClIs with rpoB mutations were resistant to RIF according to phenotypic tests.

**Table 4:** Loci and types of mutations conferring RIF resistance in MTB clinical isolates belonging to different genotypes

Loci and types of mutations	Number of MTB isolates with rpoB mutations from HIV - patients						Number of MTB isolates with rpoB mutations from HIV+ patients				
	BEIJING			LAM	Ural, X1, Haarlem	TOTAL	BEIJING			LAM, Ural	TOTAL
	TOTAL	B0 / W148	Other				TOTAL	B0 / W148	Other		
rpoB 507 Del	0	0	0	0	0	**0**	0	0	0	0	**0**
rpoB 511, Leu511Pro	10	2	8	0	0	**10**	1	0	1	1	**2**
rpoB 511, Leu511Arg,	0	0	0	0	0	**0**	0	0	0	0	**0**
rpoB 512 Ser512Thr	0	0	0	0	1	**1**	0	0	0	0	**0**
rpoB 512 Ser512Arg,	0	0	0	0	0	**0**	0	0	0	0	**0**
rpoB 513 Gln513Lys; Gln513Gly	0	0	0	0	0	**0**	0	0	0	0	**0**
rpoB 516 Asp516Tyr	7	1	6	1	1	**9**	0	0	0	0	**0**
rpoB 516 Asp516Gly	0	0	0	0	0	**0**	0	0	0	0	**0**
rpoB 516 Asp516Val	1	0	1	3	0	**4**	0	0	0	0	**0**
rpoB 516 Asp516Glu	0	0	0	0	0	**0**	0	0	0	0	**0**
rpoB 516 Asp516Ala	6	1	5	0	0	**6**	1	0	1	0	**1**
rpoB 522 Ser522Leu	0	0	0	1	0	**1**	0	0	0	0	**0**
rpoB 526 His526Tyr	2	1	1	0	0	**2**	0	0	0	0	**0**
rpoB 526 His526Asn	1	0	1	5	0	**6**	0	0	0	1	**1**
rpoB 526 His526Leu	1	0	1	0	0	**1**	0	0	0	0	**0**
rpoB 526 His526Pro	0	0	0	1	0	**1**	0	0	0	0	**0**
rpoB 526 His526Arg; His526Asp; His526Cys; His526Glu; His526Gln; His526Pro	0	0	0	0	0	**0**	0	0	0	0	**0**
**rpoB 531 Ser531Leu**	**153**	**54**	**99**	**12**	**2**	**167**	**51**	**25**	**26**	**0**	**51**
rpoB 531 Ser531Asn; Ser531Cys; Ser531Trp	0	0	0	0	0	**0**	0	0	0	0	**0**
rpoB 533 Leu533Pro	7	6	1	0	0	**7**	1	1	0	6	**7**
**TOTAL**	188	65	123	23	4	**215**	54	26	28	8	**62**

0 :- loci and types of mutations that were tested but not revealed

Beijing genotype prevailed among RIF resistant ClIs: 188 ClIs from HIV- patients of 215 tested (PR = 87,4%, CI95% = 4,4%) and 54 ClIs from HIV+ patients of 62 tested (PR = 87,1%, CI95% = 8,7%), the difference between HIV- and HIV+ groups of ClIs was not significant (*p* = 1,0). PR of B0/W148 ClIs from HIV- patients was significantly lower than of other Beijing ClIs: 65 and 123 ClIs of all 215 ClIs, correspondingly (PR = 30,2%, CI95% = 6,1% and PR = 57,2% CI95% = 6,6%, *p* < < 0,0001). No significant difference for PRs B0/W148 and other Beijing ClIs was revealed in ClIs from HIV+: 25 and 26 of 62(PR = 40,3% CI95% = 12,2% and PR = 41,9%, CI95% = 12,3%, *p* = 1,0). Other genotypes detected in HIV- group of ClIs were LAM (23 ClIs, PR = 10,7%, CI95% = 4,1%) and minor ClIs (Ural-2 ClIs, X1 and Haarlem − 1 isolate each, total number 4, PR = 1,9%, CI95% = 1,8%). In HIV+ group minor genotypes were LAM (2 ClIs, PR = 3,2%, CI95% = 4,4%), and Ural (6 ClIs, PR = 9,7%, CI95% = 7,4%),

Mutations in ClIs from HIV- patients were detected in 7 loci, total number of types of mutations - 12. There were three mutations substituting Aspartic acid by Tyrosine, Valine, and Alanine in locus rpoB516, four mutations substituting Histidine by Asparagine, Proline, Leucine, Tyrosine in locus rpoB526. In four other loci there were only one type of mutations detected in each: rpoB511 – substitution of Leucine by Proline, rpoB512 – Serine by Threonine, rpoB522 – Serine by Leucine, rpoB531 - Serine by Leucine, rpoB533 -Leucine by Proline (Table 4).

Mutations in ClIs from HIV+ patients 5 types of mutations were detected in 5 loci: rpoB511- substitution of Leucine by Proline, rpoB516 – Aspartic acid by Alanine, rpoB526 – Histidine by Asparagine, rpoB531 - Serine by Leucine, rpoB533 -Leucine by Proline.

No mutations were revealed in loci rpoB507 and rpoB513, neither in ClIs from HIV- patients, nor in ClIs from HIV+ patients (Table 4).

Ser531Leu mutation dominated among all rpoB mutations both in ClIs from HIV- (188 of all 215 resistant ClIs to RIF, PR = 87,4%, CI95% = 4,4%) and from HIV+ − 51 of 62 ClIs (PR = 82,3%, CI95% = 9,5%). Difference between these two groups is not statistically significant (*p* = 0,6). The mutation prevailed in Beijing ClIs both in HIV- (PR = 91,6%, CI95% = 4,2% in Beijing ClIs and PR = 8,4%, CI95% = 4,2% other genotypes’ ClIs) and HIV+ (51 Beijing ClIs and 0 other genotypes’ ClIs) groups, *p* < 0,001.

Leu533Pro mutation was more frequent in HIV+ ClIs group than in HIV-group: 7 ClIs in the HIV- group (PR = 3,3%, CI95% = 2,4%) and 7 in HIV+ group (PR = 11,3%, CI95% = 7,9%), the difference is statistically significant (*p* = 0,004).

Among ClIs from HIV- group of Beijing genotype 10 ClIs with mutations in rpoB511 locus (Leu511Pro, PR = 4,7%, CI95% = 2,8%), and 5 ClIs with mutations in rpoB516 locus (Asp516Ala, PR = 2,3%, CI95% = 2,0%) were detected. These mutations were not detected in ClIs of other genotypes. In HIV+ ClIs no rpoB511, Leu511Pro mutation was detected, rpoB516 Asp516Ala mutation was detected in only one isolate belonging to other than B0/W148 Beijing subclone.

### Resistance to EMB

Total number of ClIs from HIV- patients with embB mutations was 198, and of ClIs from HIV+ patients - 66. Mutations in embB gene were only detected in EMB resistant ClIs (Table 5). However, we identified 42 ClIs from HIV- patients and 5 ClIs from HIV+ patients as resistant to EMB by DST but did not detect mutations in their embB genes.

**Table 5:** Loci and types of mutations conferring EMB resistance in MTB clinical isolates belonging to different genotypes

Loci and types of mutations	Number of MTB isolates with embB mutations from HIV - patients						Number of MTB isolates with embB mutations from HIV+ patients				
	BEIJING			LAM	Ural, T1, T3, Haarlem	TOTAL	BEIJING			LAM, Ural	TOTAL
	TOTAL	B0 / W148	Other				TOTAL	B0 / W148	Other		
**embB 296** Asn296His	15	5	10	2	3	**20**	9	9	0	0	**9**
**embB297** Ser297Ala	0	0	0	0	0	**0**	1	1	0	0	**1**
**embB306** Met306Leu	**21**	**8**	**13**	**4**	**3**	**28**	**10**	**4**	**6**	**0**	**10**
**embB306,** Met306Val	**35**	**11**	**24**	**8**	**2**	**45**	**27**	**12**	**15**	**3**	**30**
**embB309** Val309Phe	5	1	4	2	3	**10**	0	0	0	0	**0**
**embB313** Ala313Val	0	0	0	0	0	**0**	3	0	3	0	**3**
**embB319** Tyr319Cys	0	0	0	1	0	**1**	0	0	0	0	**0**
**embB319** Tyr319Ser	2	0	2	0	0	**2**	0	0	0	0	**0**
**embB319** Tyr319Asp	0	0	0	0	0	**0**	0	0	0	0	**0**
**embB328** Asp328Tyr;Asp328Gly	0	0	0	0	0	**0**	0	0	0	0	**0**
**embB354** Asp354Ala	20	3	17	2	1	**23**	5	0	5	0	**5**
**embB378** Glu378Ala	0	0	0	0	0	**0**	2	0	2	0	**2**
**embB406** Gly406Ala	46	43	3	0	0	**46**	2	0	2	0	**2**
**embB406** Gly406Asp	3	3	0	0	1	**4**	0	0	0	0	**0**
**embB406** Gly406Ser	1	1	0	0	0	**1**	2	0	2	0	**2**
**embB406** Gly406Cys	0	0	0	0	0	**0**	0	0	0	0	**0**
**embB497** Gln497Lys	5	5	0	0	1	**6**	0	0	0	0	**0**
**embB497** Gln497Pro	0	0	0	0	0	**0**	0	0	0	0	**0**
**embB497** Gln497Arg	12	0	12	0	0	**12**	2	0	2	0	**2**
**TOTAL**	**165**	**80**	**85**	**19**	**14**	**198**	**63**	**26**	**37**	**3**	**66**

0 :- loci and types of mutations that were tested but not revealed

Beijing genotypes ClIs prevailed among EMB resistant ClIs with mutations in EMB (EMB resistant further on): 165 ClIs from HIV- patients of 198 tested (PR = 83,3%, CI95% = 5,0%) and 63 ClIs from HIV+ patients of 66 tested (PR = 95,5%, CI95% = 5,0%), the difference between PR of ClIs from HIV- and HIV+ is statistically significant *p* = 0,007. Other genotypes detected in HIV-group of ClIs were LAM (19 ClIs, PR = 9,6%, CI95% = 4,1%) and minor genotypes: Ural − 10, T1 and Haarlem – one isolate each, T3 – two ClIs (total number ClIs of minor genotypes– 14, PR = 7,1%, CI95% = 3,6%). There was no significant difference in PRs of B0/W148 and other Beijing ClIs from HIV- patients: 80 ClIs (PR = 48,5%, CI95% = 7,6%) and 85 (PR = 51,5%, CI95% = 7,6%) of 165 Beijing ClIs, correspondingly, *p* = 0,3, and in ClIs from HIV+ patients: 35 B0/W148 (PR = 55,6%, CI95% = 12,3%), and 28 other Beijing (PR = 44,4%, CI95% = 12,3%), of 63 Beijing ClIs, *p* = 1,6.

Mutations in ClIs from HIV- patients were detected in seven loci of embB, total number of types of mutations was 12. In locus embB306 two mutations substituting Methionine by Leucine and Valine, in locus embB319 – two types of mutations substituting Tyrosine by Cysteine, and Serine, embB406 – Glycine by Alanine, Aspartic acid and Serine, embB497 – Glutamine by Lysine and Arginine. In three other loci there were only one type of mutations detected in each: at embB296 – substitution of Asparagine by Histidine, at embB309 – Valine by Phenylalanine and at embB354 -Asparagine by Alanine (Table 5).

In ClIs from HIV+ patients eight types of mutations at five loci were detected: embB296 substitution of Asparagine by Histidine, embB297 – Serine by Alanine, embB306- two mutations substituting Methionine by Leucine and Valine, embB313 – Alanine by Valine, embB354 - Aspartic acid by Alanine, embB378 - Glutamic acid by Alanine, embB378- Glutamic acid by Ala, embB406 – Glycine by Alanine and Serine, embB497 Glutamine by Arginine (Table 5).

Mutations in locus embB306 (two types of mutations) were most frequent among all embB mutations both in ClIs from HIV- (73 of all 198 ClIs resistant to EMB, PR = 36,9%, CI95% = 6,7%) and from HIV+ patients - 40 of 66 ClIs (PR = 60,6%, CI95% = 11,8%). PR of mutations in this locus in ClIs from HIV+ patients was higher than from HIV- patients, the difference is statistically significant (*p* = 0,0001). PRs did not differ significantly: for Met306Leu in HIV-group (28 of 198 ClIs) it was 14,1% (CI95% = 4,9%) and in HIV+ group (10 of 72 ClIs) – 13,9% (CI 95% = 8,0%), *p* = 1,0. They differed for Met306Val: its PR in HIV- group (45 of 198 ClIs) was 22,7% (CI95% = 5,8%) and in HIV+ group (30 ClIs of 66) -45,5% (CI 95% = 12,0%), the difference is statistically significant (*p* < < 0,0001).

PRs of mutations in locus embB306 were significantly higher in Beijing genotype ClIs than of other genotypes, both in HIV- and HIV+: in HIV-group, PR = 76,7%, CI95% = 9,7% and PR = 23,3, CI95% = 3,3%, correspondingly, (*p* < < 0,0001); in HIV+ group PR = 95,5%, CI95% = 5,0% and PR = 4,5%, CI95% = 5,0%, correspondingly, (*p* < < 0,0001). The difference of PRs of non-Beijing genotypes in HIV- and HIV+ ClIs was also statistically significant (*p* < < 0,02).

No mutations were revealed in loci embB328 Asp328Tyr, neither in ClIs from HIV- patients, nor in from HIV+ patients (Table 4). Mutations in locus emb406 (Gly406Ala) were frequently detected in ClIs from HIV-patients (46 ClIs of 198, PR = 24,9%, CI95% = 6,2%), but not in HIV+ group (2 ClIs of 72, PR = 2,8%, IC95% = 3,8%), the difference is significant, *p* = 0,00002. The mutation was mainly detected in ClIs belonging to B0/W148 Beijing genotype (43 of all 80 EMB resistant ClIs of this genotype, PR = 53,8%, CI95% = 10,9%).

Mutations in loci: embB309 (Val309Phe) and embB319 (Tyr319Cys and Tyr319Ser) were detected in ClIs from HIV- patients only. Alternatively, mutations in loci embB297 (Ser297Ala), embB313 (Ala313Val) and embB378 (Glu378Ala) were detected only in in ClIs from HIV+ patients.

The highest PR of other types of mutations was for embB354 Asp354Ala (11,6%, CI95% = 4,8) in HIV- group ClIs and for embB296 Asn296His (12,5%, CI95% = 7,7%) in HIV+ group ClIs. PRs of other types of mutations were significantly lower.

### Resistance to fluoroquinolones

In this study resistance to Ofl was chosen as a marker of resistance to FQ, although some of ClIs resistant to Ofl in the studied groups of clinical ClIs retained susceptibility to Levofloxacin and/or Moxifloxacin. The choice of Ofl resistance gave the possibility to enlarge the group of FQ resistant ClIs. In this group all ClIs bear mutations in gyrA and gyrB genes. We were not aimed to analyze associations of particular mutations with resistance to different FQ.

Total number of FQ resistant ClIs from HIV- patients was 73, and from HIV+ patients - 24. Mutations in gyrA and gyrB gene were detected (Table 5). All ClIs with gyrA and/or gyrB genes mutations were resistant to Ofl according to DST. In the group of ClIs from HIV-patients gyrA mutations were detected in 50 XDR ClIs (PR = 74,6% CI95% = 10.4%), in 17 preXDR ClIs (PR = 25,4% CI95% = 10,4%). Four XDR ClIs bear both gyrA and gyrB mutations (all Asp94Gly- Ser95Thr, Asn538Asp). One XDR isolate belonging to other than B0/W148 Beijing genotype had two gyrA mutations (Ala90Val-Ser91Pro, Asp94Asn- Ser95Thr) and gyrB mutation (Ala543Val). In loci and mutations prevalence analysis each of these mutations were considered as mutations in separate ClIs.

In the HIV+ patients’ group of ClIs gyrA mutations were detected in 24 ClIs, four in monoresistant, 13- in preXDR and 10 in XDR ClIs. No gyrB mutations or ClIs with double mutations in gyrA and gyrB gene were detected in this group.

Beijing genotype prevailed in ClIs with gyrA mutations: 57 ClIs of 67 of HIV-group (PR = 85,1% CI95% = 8,5%) and 22 of 25 in HIV+ group (PR = 88,8% CI95% = 12,7%). PR of gyrA bearing ClIs was significantly lower among B0/ W148 ClIs than among ClIs of other Beiging genotypes in HIV- group – 21 (PR = 31,3% CI95% = 11,1%) and 35 (PR = 52,2%, CI95% = 12,0%), *p* = 0,014; and was higher in HIV+ group- 14 (PR = 56,0% CI95% = 19,5%) and 8 (PR = 32,0% CI95% = 18,3%),correspondingly, *p* = 0,15. Non-Beijing gyrA mutant ClIs from - HIV- group were LAM (7 ClIs, PR = 11,1% CI95% = 7,8%), Ural (two ClIs) and X1-one isolate (for both genotypes PR = 4,8% CI95% = 5,3). In HIV+ group of ClIs non-Beijing with gyrA mutations was Ural only, (3 ClIs, PR = 12,0% CI95% = 12,7%).

Beijing genotype prevailed in ClIs of HIV-group with gyrB mutations: 10ClIs of 11 (PR = 90,9% CI95% = 17,0%). PR of ClIs with gyrB mutations were significantly lower in B0/ W148 ClIs compared with ClIs of other Beijing genotypes - two ClIs (PR = 18,2% CI95% = 22,8%) and 8 (PR = 72,7% CI95% = 26,3%), correspondingly, *p* = 0,03. Only one isolate with mutation gyrB539 Thr539 Ile belonging to Ural genotype was identified.

There were four loci with gyrA mutations in HIV- group of ClIs (gyrA70, gyrA90–91, gyrA94–95, gyrA102) and in HIV+ group of ClIs (gyrA80, gyrA90–91, gyrA94–95, gyrA102). GyrB mutations were detected in loci gyrB486, gyrB500, gyrB538, gyrB539, gyrB543. No gyrA mutations were detected in loci gyrA74, gyrA80 and gyrA88 in ClIs from HIV- patients and gyrA74 and gyrA88 in ClIs from HIV+ patients; no gyr B mutations were detected in loci gyrB485, gyrB509, gyrB533, gyrB540.

In ClIs from HIV- patients gyrA mutations were mainly located in gyrA90–91 (Ala90Val-Ser91Pro and Ala90Gly-Ser91Pro), 15 ClIs, PR = 22,4% CI95% = 10,0%, and gyrA94–95 (Asp94Ala -Ser95Thr, Asp94Gly- Ser95Thr, Asp94His- Ser95Thr, Asp94Asn- Ser95Thr), 46 ClIs, PR = 65,7% CI95% = 11,4%. Most prevailing types of mutations were Asp94Ala -Ser95Thr 12 ClIs (PR = 17,9% CI95% = 9,2%), Asp94Gly- Ser95Thr 18 ClIs (PR = 26,9% CI95% = 10,6%) and Asp94Asn- Ser95Thr 10 ClIs (PR = 14,9% CI95% = 8,5%) (Table 6).

**Table 6:** Loci and types of mutations conferring FQ resistance in MTB clinical isolates belonging to different genotypes

Loci and types of mutations	Number of MTB isolates with gyrA and gyrB mutations from HIV - patients						Number of MTB isolates with gyrA and gyrB mutations from HIV+ patients				
	BEIJING			LAM	Ural, X1	TOTAL	BEIJING			Ural	TOTAL
	TOTAL	B0 / W148	Other				TOTAL	B0 / W148	Other		
**gyrA70** His70Arg	1	0	1	0	0	**1**	0	0	0	0	**0**
**gyrA74** Ala74Ser	0	0	0	0	0	**0**	0	0	0	0	**0**
**gyrA80**Thr80Ala	0	0	0	0	0	**0**	1	0	1	0	**1**
**gyrA 88** Gly88Ala; Gly88Cys	0	0	0	0	0	**0**	0	0	0	0	**0**
**gyrA 90-91** Ala90Val-Ser91Pro	6	0	6	0	1	**7**	**5**	**4**	**1**	**1**	**6**
**gyrA 90-91** Ala90Gly-Ser91Pro	8	8	0	0	0	**8**	0	0	0	0	**0**
**gyrA 94-95** Asp94Ala -Ser95Thr	**11**	**7**	**4**	**1**	**0**	**12**	**2**	**2**	**0**	**1**	**3**
**gyrA 94-95** Asp94Gly- Ser95Thr	**16**	**0**	**16**	**2**	**0**	**18**	**8**	**6**	**2**	**0**	**8**
**gyrA 94-95** Asp94His- Ser95Thr	2	0	2	2	0	**4**	0	0	0	0	**0**
**gyrA 94-95** Asp94Asn- Ser95Thr	**9**	**4**	**5**	**0**	**1**	**10**	4	2	2	1	**5**
**gyrA 94-95** Asp94Val- Ser95Thr	0	0	0	0	0	**0**	0	0	0	0	**0**
**gyrA 94-95** Asp94Tyr- Ser95Thr	1	0	1	0	1	**2**	1	0	1	0	**1**
**gyrA 102** Pro102His	3	2	1	2	0	**5**	1	0	1	0	**0**
**Total gyrA**	**57**	**21**	**36**	**7**	**3**	**67**	**22**	**14**	**8**	**3**	**25**
**gyrB 485** Arg485His; Arg485Leu	0	0	0	0	0	**0**	0	0	0	0	**0**
**gyrB 486** Ser486Phe	2	1	1	0	0	**2**	0	0	0	0	**0**
**gyrB 500** Asp500Asn	2	0	2	0	0	**2**	0	0	0	0	**0**
**gyrB 500** Asp500His; Asp500Ala	0	0	0	0	0	**0**	0	0	0	0	**0**
**gyrB 509** Gly509Cys; Gly509Ala	0	0	0	0	0	**0**	0	0	0	0	**0**
**gyrB 525** Ile525Leu	0	0	0	0	0	**0**	0	0	0	0	**0**
**gyrB 533** Asp533Ala	0	0	0	0	0	**0**	0	0	0	0	**0**
**gyrB 538** Asn538 Asp	1	0	1	0	0	**1**	0	0	0	0	**0**
**gyrB 538** Asn538Tyr; Asn538Lys; Asn538Thr	0	0	0	0	0	**0**	0	0	0	0	**0**
**gyrB 539** Thr539 Ile	0	0	0	0	1	**1**	0	0	0	0	**0**
**gyrB 539** Thr539 Asn	0	0	0	0	0	**0**	0	0	0	0	**0**
**gyrB 539** Thr539Pro	1	0	1	0	0	**1**	0	0	0	0	**0**
**gyrB 540** Glu540Asp	0	0	0	0	0	**0**	0	0	0	0	**0**
**gyrB 543** Ala543Thr	2	0	2	0	0	**2**	0	0	0	0	**0**
**gyrB 543** Ala543Val	2	1	1	0	0	**2**	0	0	0	0	**0**
**Total gyrB**	**10**	**2**	**8**	**0**	**1**	**11**	**0**	**0**	**0**	**0**	**0**

0 :- loci and types of mutations that were tested but not revealed

In ClIs from HIV+ patients Ala90Val-Ser91Pro mutation was detected in six ClIs (PR = 24,0% CI95% = 16,7%), Asp94Ala -Ser95Thr and Asp94Gly- Ser95Thr in 11 ClIs (PR = 44,0% CI95% = 19,5%).

No dominating loci or type of mutations in gyrB gene were observed (Table 6).

### Resistance to aminoglycosides

Although the latest WHO clinical guidelines and recommendations of 2021 exclude injectable drugs Kn and Cap from the chemotherapy regimens and DST [18, 19], phenotypic DST for these drugs were included in this study as it was done mainly in 2018–2019. However, we found different mutations spectra conferring to Kn, Ami, Cap, like in other researches [20, 21]. Total number of ClIs resistant to Kn or Ami from HIV- patients was 97, and of ClIs from HIV+ patients - 24. Mutations in regulatory regions of eis and rrs genes were detected (Table 6). All ClIs with mutations in eis and rrs genes were resistant to Kn according to DST, five were also resistant to Ami and Cap. All mutants in rrs gene both in HIV- and HIV+ groups of ClIs were resistant to Ami and Cap.

In the group of ClIs from HIV-patients mutations in eis gene were detected in 70 ClIs (PR = 72,2% CI95% = 8,9%). Mutations in rrs gene were detected in 27 ClIs (PR = 27,8% CI95% = 8,9%).

In the group of ClIs from HIV+ patients mutations in eis gene were detected in 15 ClIs (PR = 62,5% CI95% = 19,4%).. Mutations in rrs gene were detected in 9 ClIs (PR = 37,5% CI95% = 19,4%).

Beijing genotype prevailed in ClIs from HIV- patients, both in those with eis mutations (52 of 70, PR = 74,3% CI95% = 10,2%) and with rrs mutations (26 of 27, PR = 96,3% CI95% = 7,1%). B0/W148 genotype PRs were lower both in eis and rrs bearing ClIs (15 ClIs with eis mutations, PR = 21,4% CI95% = 9,6%, 9 ClIs with rrs mutations PR = 33,3% CI95% = 17,8%) than PR for ClIs of other Beijing genotypes (37 ClIs with eis mutations, PR = 74,3% CI95% = 10,2%, 17 ClIs with rrs mutations PR = 63,0% CI95% = 18,2%). Differences were statistically significant for ClIs with eis (*p* = 0,0002) and with rrs mutations (*p* = 0,05). Non-Beijing genotypes in eis bearing ClIs were LAM (15 ClIs, PR = 21,4% CI95% = 9,6%) and Ural (3 ClIs, PR = 4,3% CI95% = 4,7%); in ClIs with rrs there was only one isolate of genotype, other than Beijing – Ural genotype (Table 7).

**Table 7:** Loci and types of mutations conferring aminoglycosides and capreomycin resistance in MTB clinical isolates belonging to different genotypes

Loci and types of mutations	Number of MTB isolates with eis and rrs mutations from HIV – patients						Number of MTB isolates with eis and rrs mutations from HIV+ patients				
	BEIJING			LAM	Ural	TOTAL	BEIJING			Ural	TOTAL
	TOTAL	B0 / W148	Other				TOTAL	B0 / W148	Other		
**eis** g10a	**21**	**7**	**14**	**3**	**1**	**25**	6	4	2	1	**7**
**eis** g10c	0	0	0	0	0	**0**	0	0	0	0	**0**
**eis** c12t	4	2	2	4	1	**9**	1	1	0	1	**2**
**eis** a13g	0	0	0	0	0	**0**	0	0	0	0	**0**
**eis c**14t	12	4	8	1	1	**14**	4	2	2	0	**4**
**eis g**37t	**15**	**2**	**13**	**7**	**0**	**22**	2	0	2	0	**2**
**Total eis**	**52**	**15**	**37**	**15**	**3**	**70**	**13**	**7**	**6**	**2**	**15**
**rrs** a1401g	**26**	**9**	**17**	**0**	**0**	**26**	**8**	**0**	**8**	**0**	**8**
**rrs c**1402a	0	0	0	0	0	**0**	0	0	0	0	**0**
**rrs** c1402t	0	0	0	1	0	**1**	0	0	0	0	**0**
**rrs g**1484t	0	0	0	0	0	**0**	1	0	1	0	**1**
**Total rrs**	**26**	**9**	**17**	**1**	**0**	**27**	**9**	**0**	**9**	**0**	**9**

0 :- loci and types of mutations that were tested but not revealed

In the group of ClIs from HIV+ patients PR of Beijing genotype ClIs was significantly higher than of other genotypes, presented by Ural only: 22 Beijing ClIs of 24, PR = 91,7% CI95% = 11,1%, and 2 Ural ClIs, PR = 8,3% CI95% = 11,1%. ClIs with rrs mutations had only Beijing genotype (Table 7).

Eis mutations were promotor-region mutations in loci eis10 (Gly10Ala), eis12 (Cys12Thr), eis 14 (Cys14Thre) and eis 37 (Gly37Thre), both in ClIs from HIV- and HIV+ patients. Mutation eis10 Gly10Ala was detected in 25 ClIs from HIV- patients (PR = 35,7% CI95% = 11,2%) and in seven ClIs from HIV+ patients (PR = 46,7% CI95% = 25,2%). Mutation Gly37Thr in locus eis 37 was detected in 22 ClIs from HIV- patients PR = 31,4% CI95% = 10,9%) and in only two ClIs from HIV+ patients (PR = 13,3% CI95% = 17,2%). Two ClIs with Gly10Ala and tree Cys14Thre were resistant to Kn and, additionally to Ami and Cap.

Prevailing mutation in promoter of rrs gene was Ala1401Gly, both in ClIs from HIV- and HIV+ patients. One isolate from HIV- patients with mutation rrs Cys1402Thr and one from HIV+ patients rrs Gly1484Thr were revealed.

No ClIs with mutations in locus eis13, were found neither from HIV-, nor from HIV+ patients.

## Discussion

MTB genotypes and mutations’ landscapes in many regions of the world had been described during the last 30 years [22, 23]. Genotypes typical for the Russian Federation revealed with spoligotyping, MIRU-VNTR analysis and whole genome sequencing were also described in many publications [8, 22, 23]. Extended research is in progress today to reveal MICs of anti-TB drugs’ associations with particular mutations, directed to development of reliable DST and molecular tests highly correlated with efficiency of chemotherapy with a particular drug [24]. In our study we used commercial test-system, designed to reveal most frequent in the Russian Federation genotypes and mutations, associated with DR in MTB, and conventional DST methods. Despite certain limitations of the methods used we had revealed certain regularities in genotypes, types of mutations conferring DR of MTB and HIV status of patients – sample sources.

### Genotypes and DR profiles

In our research Beijing genotype (including 82 B0/W148 Beijing) ClIs prevailed in the group from HIV- patients (64,7%) and from HIV+ patients (74,4%) – the difference is statistically significant. Beijing genotype was the most frequent genotype in different Russian regions according to other publications [22, 23]. Beijing subclone B0/W148 was assigned as one of the most widely distributed clusters in the Russian Federation and a “successful” clone in other studies [25–28]. However, it was not the most frequent genotype in the groups of ClIs we studied: PRs of B0/W148 were 19,0% in ClIs from HIV- patients and 20,2% in ClIs from HIV+ patients. Other Beijing ClIs comprised 45,7% and 54,2%, correspondingly.

PRs for non-Beijing genotypes were in ClIs from HIV- and HIV+ patients: LAM – 17,2% and 7,8% and Ural – 8,1% and 14,7%, correspondingly. Prevalence of LAM genotype was lower than in earlier studies of genotypes’ distribution in Russia:17,2% and 31,0% [28, 29]. PR of Ural genotype ClIs correlates with 7% of Ural strains in the MTB population of the Russian Federation, reported earlier [30]. In ClIs from HIV+ patients higher PR for Beijing and Ural genotypes were observed, compared to ClIs from HIV- patients (*p* = 0,04), rate of LAM isolate was lower in ClIs from HIV- patients (PR was 8,1% and 14,7%, *p* = 0,008). In ClIs from HIV-patients 10,3% of ClIs belonged to 7 minor genotypes (LAM1, LAM9, T1, T1Rus, T2, T3, T4, EAL, Haarlem). In ClIs from HIV+ patients only LAM9 and EAL minor ClIs were detected (3,2%).

Beijing genotype ClIs were revealed in ClIs of all DR profiles, both in the groups isolated from HIV- and HIV+ patients. PRs of Beijing genotypes in MDR-XDR ClIs were higher than in susceptible ClIs, both in ClIs from HIV- and HIV+ patients: 86,6% and 88,5% in drug resistant ClIs and 36,6% and 63,7%, correspondingly. Differences in PRs of Beijing phenotypes in susceptible and MDR-XDR ClIs were statistically significant (for ClIs from HIV- patients *p* < < 0, 0001 and for ClIs from HIV+ patients *p* = 0,0002, correspondingly).

Beijing genotype dominated in ClIs with mutations conferring resistance to antituberculosis drugs, PRs were for katG 83,4 and 100%, for inhA 72,1% and two of five ClIs from HIV- and HIV+ patients, correspondingly. PRs mutants of Beijing phenotype were for rpoB 87,4% and 87,1%, for embB – 83,3% and 93,5%, for gyrA 85,1% and 88,8%, for gyrB 90,1% and zero (no gyr B mutants detected), for eis – 74,3% and 91,7%, rrs- 96,3 and 100% of ClIs from HIV- and HIV+ patients, correspondingly.

B0/W148 subclone of Beijing genotype was identified in rare compared to other Beijing subclones in ClIs with majority of mutations conferring resistance to antituberculosis drugs, PRs were for katG – 30,3% and 53,6%, inhA – 7,0% and 65,1%, rpoB 30,2% and 57,2%, gyrA 31,3% and 53,7%, eis – 21,4% and 74, 4%, rrs 33,3% and 63,05 in B0/W148 subclone and other Beijing subclones ClIs from HIV- patients, correspondingly. There were no differences in PRs in ClIs from HIV+ patients for rpoB, gyrB, and in ClIS from both HIV+ and HIV- patients for embB.

Prevalence of Beijing phenotypes in MDR-XDR profiles agrees with the hypothesis of higher mutation rates in these types of strains [9, 15, 23, 31–33]. However, in our study frequencies of Beijing genotype in ClIs susceptible to all drugs both in groups from HIV- and HIV+ patients were also high: 37,7% and 63,7%, correspondingly. It implies that Beijing genotype may not be the only factor of DR development, but rather cooperate with other factors, such as, for example, individual characteristics of the host immunity. B0/W148 genotype was not detected in susceptible ClIs neither from HIV- nor HIV+ patients. In MDR-XDR ClIs from HIV- patients its PR was significantly lower than of other Beijing genotypes (29,1% and 55,3%, correspondingly, *p* = 0,0002), no significant difference was revealed in these two groups of ClIs from HIV+ patients.

PRs of non-Beijing genotypes ClIs (LAM, LAM1, LAM9, URAL, and minor genotypes) were significantly higher in susceptible ClIs from HIV- and HIV+ patients, than in MDR-XDR groups.

### Mutations conferring resistance to INH

In this study mutations conferring INH resistance were detected in katG, inhA and ahpC genes. In 9,0% of all INH resistant ClIs from HIV- patients’ mutations in both katG and inhA genes were detected. All double mutants were of Beijing genotype (other than B0/W148 Beijing genotypes). No double mutants were detected in the HIV+ group. All ClIs with mutations, conferring resistance to INH (except one isolate with sole ahpC C12T mutation) were resistant to corresponding drugs in DST.

Only one isolate from HIV- group with mono ahpC C12T mutation was identified, and this isolate was susceptible to all drugs. Gene ahpC is coding for Alkyl- hydroperoxide reductase C and is a part of ahpC-OxiR regulon, coding for enzymes responsible for the detoxification of reactive oxygen. The system is normally inactive in MTB cells and is activated by mutations inducing its expression when inactivation of catalase-peroxidase by katG mutation occur, ahpC mutations are compensatory mutations for mutations in katG gene [24, 34, 35]. As compensatory mutations ahpC mutations are often detected in katG mutants and so are associated with INH resistance [24, 35–38].

Analysis of prevalence INH resistance conferring mutations revealed patients Beijing ClIs.

In 254 ClIs from HIV- patients 83,1% harbored mutations in katG gene and 16,9% in inhA gene. All katG mutations in ClIs from HIV- patients were in four loci: three types of mutations at katG315 (substitution of Serine by Threonine, Arginine and Glycine), at kat328 - substitution of Tryptophan by Cysteine and at katG335- substitution of Isoleucine by Valine. Variability of types of mutations in katG gene was lower in HIV+ group of ClIs: only two types of mutations were detected, both in katG315 loci. Most frequent mutation in HIV- and HIV+ group of ClIs was Ser315Thr (PR = 94,8% and 98,7%, correspondingly) associated with non-B0/W148 Beijing genotypes.

All detected mutations in inhA loci were in the inhA promotor region [30, 35]. The most frequent mutation type in ClIs from HIV- and HIV+ patients was T15A (PR = 81,4% in HIV-group of ClIs and 100% in HIV+ ClIs). This type of mutations was associated with other than B0/W148 Beijing genotypes in ClIs from HIV- patients (PR = 65,7%). Number of HIV+ ClIs with inhA mutations were only five, no significant differences were revealed. Other investigators published similar results on prevalence of katG and inhA mutations in INH resistant MBT ClIs, and locus katG235 as the most frequent locus of mutations [35, 38–40]. Studies of mutations associated with INH resistance in MTB in Kyrgyz Republic also revealed prevailing mutations katG Ser315Thr (88.6%) and the only one mutation in inhA region was T15A [40].

Polymorphism in katG gene was higher in Beijing ClIs in HIV- group compared to other genotypes, and in HIV-group compared to HIV+ group.

### Mutations conferring resistance to RIF

Mutations in rpoB gene were the only detected, according to the design of test system used.

In ClIs from HIV- patients we had detected 12 types of mutations in seven loci in rpoB gene, in ClIs from HIV+ patients – five types of mutations in five loci. Types of mutations detected in ClIs from HIV- and HIV+ patients were: Leu511Pro, Asp516Ala, His526Asp, Ser531Leu, Leu533Pro. Mutations in loci rpoB512 (Ser512Thr) rpoB516 (Asp516Val and Asp516Tyr), rpoB522 (Ser522Leu), rpoB526 (His526Pro, His526Leu, His526Tyr) were detected in ClIs from HIV-patients only. Leu533Pro mutation was significantly more frequent in HIV+ ClIs group than in HIV-group (*p* = 0,004).

Most frequent type of mutation in ClIs from HIV- and HIV+ patients was Ser531Leu - (PR = 87,4% and PR = 82,3%, correspondingly). Similar results were reported in other publications [14, 39]. No significant differences in rates of this type of mutation in Beijing and other genotype groups of ClIs were observed. Mutations Leu511Pro (PR = 4,7%) and Asp516Ala (PR = 2,3%) were detected in ClIs of Beijing genotype only.

According to the test system facilities all mutations detected were in the region of rpoB511-rpoB533 (23-bp long), which is a part of the rpoB 81-bp core region [41] – the RIF resistance-determining region (RRDR). Ser531Leu mutation dominated in RIF resistant mutants in the group of ClIs we had studied, as in ClIs from Kirgizia [40]. This mutation was associated with high MICs for RIF and Rifabutin [24, 42]. Some of other mutations revealed in our investigations (Leu511Pro, PR = 4,3% of RIF resistant ClIs from HIV- patients and PR = 3,2% of ClIs from HIV- patients) and rare Asp516Val (4 ClIs from HIV- patients) and Ser522Leu (1 isolate from HIV- patients) were resistant to RIF in our study and were found susceptible in another [42]. However, according to WHO recommendations any mutation of RRDR, except for synonymous mutations, should be assumed to confer RIF resistance [24, 43].

### Mutations conferring resistance to EMB

Variability of loci and mutations conferring resistance to EMB was higher in ClIs from HIV- than from HIV+ patients: loci with mutations detected were seven and five, correspondingly, number of mutation’s types were 12 and eight, correspondingly.

Mutations in locus embB306 were most frequent among all embB mutations both in ClIs from HIV- (PR = 36,9%) and HIV+ patients (PR = 60,6%). Prevalence of emb306 mutations were also highlighted in other publications [24, 44]. PR of mutations in this locus in ClIs from HIV+ patients was significantly higher than from HIV- patients (*p* < < 0,0001). The difference was in PR of Met306Val: 22,7% for HIV-group of ClIs and 50,0% for HIV+ group (*p* = 0,0001). PRs of Beijing genotype ClIs with mutations in locus embB306 were significantly higher than of other genotypes ClIs, both in HIV- and HIV+: 76,7% and 23,3% correspondingly, (*p* < < 0,0001) for HIV-group and 95,5% and 4,5% in HIV+ group (*p* < < 0,0001).

In ClIs from HIV- patients’ mutation in locus emb406 (Gly406Ala) was more frequent than in HIV+ group: PRs were 24,9% and 2,8%, correspondingly, *p* = 0,00002. This mutation was detected mainly in ClIs of B0/W148 Beijing genotype (PR = 53,8% of all ClIs of HIV- group of this genotype). Mutations in loci: embB309 (Val309Phe) and embB319 (Tyr319Cys and Tyr319Ser) were detected in ClIs from HIV- patients only. Alternatively, mutations in loci embB297 (Ser297Ala), embB313 (Ala313Val) and embB378 (Glu378Ala) were detected only in in ClIs from HIV+ patients.

### Mutations conferring resistance to fluoroquinolones

Rate of gyrA mutants among ClIs from HIV- patients of B0/W148 genotype was significantly lower than of other Beijing genotypes (31,3% and 53,7%, correspondingly, *p* = 0,014). There was no significant difference between HIV+ groups of ClIs with gyrA mutations of B0/ W148 and other Beijing genotype. Non-Beijing ClIs from HIV- group with gyrA mutations were LAM, Ural and X1, in HIV+ group of ClIs one isolate was of Ural genotype only.

90,9% of gyrB mutations containing ClIs from HIV- patients were of Beijing genotype, most of them (72,7%) were of other than B0/W148 genotypes.

In ClIs from HIV- patients gyr A mutations most frequently occurred in loci gyrA 90–91 Ala90Val-Ser91Pro and Ala90Gly-Ser91Pro (PR = 22,4% for both mutations) and gyrA 94–95- Asp94Ala -Ser95Thr (PR = 17,9%), Asp94Gly- Ser95Thr (PR = 26,9%), Asp94Asn- Ser95Thr (PR = 14,9%). In ClIs from HIV+ patients most frequent mutations were Ala90Val-Ser91Pro (PR = 24,0%), Asp94Ala -Ser95Thr and Asp94Gly- Ser95Thr (PR = 44,0% both). No dominating types of mutations were in gyrB gene.

### Mutations conferring resistance to aminoglycosides and Capreomycin

We were detecting mutations in eis and rrs genes. All ClIs with mutations in these genes were resistant to Kn according to DST. All rrs mutants both from HIV- and HIV+ groups of ClIs were also resistant to Ami and Cap, only five of eis mutants from HIV-group were additionally resistant to Ami and Cap. These findings agreed with other studies’ data rrs mutants had high levels of MICs for Kn and cross-resistance to Ami and Cap, eis - moderate level of resistance to Kn and in some cases to Ami and Cap [20, 21, 24]. In the group of ClIs from HIV-patients eis mutations were detected in 70 ClIs (PR = 72,2%) and rrs mutations in 27 ClIs (PR = 27,8%). In the group of ClIs from HIV+ patients eis mutations were detected in 15 ClIs (PR = 62,5%) and rrs in 9 ClIs (PR = 37,5%).

Prevailing mutation in promoter of rrs gene was rrs Ala1401Gly, both in ClIs from HIV- and HIV+ patients. One isolate from HIV- patients with mutation rrs Cys1402Thr and one from HIV+ patients rrs Gly1484Thr were revealed. These findings agreed with other studies’ data: rrs mutations and Ala1401Gly were associated with MICs high levels for Kn [20, 21].

Eis mutations were promotor mutations in loci eis10 (Gly10Ala), eis12 (Cys12Thr), eis 14 (Cys14Thre) and eis 37 (Gly37Thre), both in ClIs from HIV- and HIV+ patients. Mutation eis10 Gly10Ala was detected in 25 ClIs from HIV- patients (PR = 35,7% CI95% = 11,2%) and seven ClIs from HIV+ patients (PR = 46,7% CI95% = 25,2%). Mutation Gly37Thr in locus eis 37 was detected in 22 ClIs from HIV- patients PR = 31,4% CI95% = 10,9%) and in only two ClIs from HIV+ patients (PR = 13,3% CI95% = 17,2%). Two ClIs with mutations Gly10Ala and tree Cys14Thr were resistant to Kn and, additionally to Ami and Cap.

Mutations in eis and rrs genes detected in our study were described in other publications as conferring different levels of resistance to Kn, Ami and Cap [20, 21, 24].

## Conclusions

Beijing genotype ClIs prevailed both in the group from HIV- patients (64,7%) and from HIV+ patients (74,4%). Beijing subclone B0/W148 Beijing was not the most frequent genotype in the groups of ClIs we studied: PRs of B0/W148 were 19,0% in ClIs from HIV- patients and 20,2% in ClIs from HIV+ patients. Other genotypes were LAM1, LAM9, T1, T1Rus, T2, T3, T4, EAL, Haarlem (10, 3%) in ClIs from HIV-patients and.

In ClIs from HIV- patients non-Beijing genotypes were: LAM – 17,2%, Ural – 8,1% and from HIV+ patients LAM 7,8%, Ural − 14,7% and 7 minor genotypes LAM1, LAM9, T1, T1Rus, T2, T3, T4, EAL, (10, 3%) and from HIV+ LAM − 7,8% and Ural − 14,7% and minor genotypes Haarlem, LAM9 and EAL (3,2. Beijing genotypes in MDR-XDR ClIs were higher than in susceptible ClIs, both in ClIs from HIV- and HIV+ patients: 86,6% and 88,5% in drug resistant ClIs and 36,6% and 63,7% in susceptible ones, correspondingly.

Prevalence of non-Beijing genotypes ClIs (LAM, LAM1, LAM9, URAL, and minor genotypes) were significantly higher in susceptible ClIs from HIV- and HIV+ patients, than in MDR-XDR groups, and higher than prevalence of Beijing genotype in susceptible ClIs from HIV- patients.

Prevailing mutations conferring resistance to INH were katG Ser315Thr and T15A and conferring RIF resistance - rpoB Ser531Leu, both in ClIs from HIV- and HIV+ patients.

Mutations in locus embB306 were most frequent among all embB mutations both in ClIs from HIV- (PR = 36,9%) and HIV+ patients (PR = 63,3%).

Prevalence of Beijing genotype ClIs with emb306 mutations was significantly higher than of other genotypes ClIs, both from HIV- and HIV+ patients. Mutation in locus emb406 (Gly406Ala), embB309 (Val309Phe) and embB319 (Tyr319Cys and Tyr319Ser) were associated with ClIs from HIV- patients only, whereas mutations embB297 (Ser297Ala), embB313 (Ala313Val) and embB378 (Glu378Ala) were detected only in in ClIs from HIV+ patients.

In ClIs resistant to Ofl most frequent were gyrA mutations in loci gyrA 90–91 and gyrA 94–95 both in ClIs from HIV- and HIV+ patients.

The dominating mutation in rrs gene was Ala1401Gly, both in ClIs from HIV- and HIV+ patients. Variability of mutations in eis gene was higher: mutations Gly10Ala, Cys12Thr, Cys14Thr and Gly37Thr were frequently detected both in ClIs from HIV- and HIV+ patients.

Spectra of genotypes and types of mutations detected in ClIs from HIV- patients were wider than in ClIs from HIV+ patients.

## Methods

### Design

Mtb ClIs were received in the NMRC PhID and URIPh laboratories from four different regions of the Russian Federation. Mtb were isolated from pulmonary specimens (mainly sputum) from newly diagnosed TB patients in 2018–2019 using liquid culturing on Bactec MGIT 960 system in laboratories of regional centers. In this study only one isolate collected from a patient before the beginning of chemotherapy were included.

Sampling of ClIs in all regions was for the purpose of external quality control, was random and targeted to adequate representation of susceptible, drug resistant (including MDR and XDR) isolates. ClIs in the samples were to be both from HIV- and HV+ patients and isolated from biological materials collected before beginning of chemotherapy, one isolate from a patient. Although these rules were followed in most of cases and considering that the only information on patients, we can operate was their HIV status (no personal data were used in this study) we cannot totally exclude clustering of isolates in exceptional cases. None of the rates presented in this manuscript could be considered as an epidemiological indicator.

Isolates were supplied with data on HIV status of patient. Total number of clinical isolates was 562, including 433 from HIV negative (HIV−) (262 isolates from Chelyabnskaya oblast, 60- from Leningradskaya, 28-from Kemerovskaya, 83 from Stavropolskiy Kray) and 129 from HIV positive (HIV+) patients (62 isolates from Chelyabnskaya oblast, 20- from Leningradskaya, 30-from Kemerovskaya, 17- from Stavropolskiy Kray). No personal patients’ data were utilized in this study.

Phenotypic drug susceptibility retesting, detection of mutations conferring drug resistance to specific drugs and genotyping of Mtb isolates had been carried out in NMRC PhID and URIPh with similar technologies.

### Drug susceptibility tests (DST)

BACTEC-MGIT 960 was used for DST [45] for Isoniazid - INH (critical concentration 0,1 mg/l), Rifampicin – RIF (critical concentration 1 mg/l), Streptomycin – S (critical concentration – 1 mg/l), Ethambutol – EMB (critical concentration-5,0 mg/l), Kanamycin – Kn (critical concentration − 2,5 mg/l), Amikacin – Amk (critical concentration 1 mg/l), Capreomycin – Cap (critical concentration 2,5 mg/l), Ofloxacin – Ofl (critical concentration 2,0 mg/l).

Isolates were characterized according to their DR profiles according to WHO recommendations [46] as:

- Susceptible– susceptible to all drugs tested.
- Mono-resistant – resistant to only one of the tested drugs.
- Poly-resistant – resistant to 2 and more drugs tested, except resistance to both INH and RIF.
- Multidrug resistance (MDR) - resistance at least to INH and RIF,
- pre-XDR - resistance at least to INH and RIF and at least to one of fluoroquinolones (FQ) or at least one of the injectable drugs (Kn, Ami, Cap),
- XDR - resistance at least to INH and RIF (MDR) and at least to one of the FQ and at least one of the injectable drugs (Kn, Amk,Cap).

Studies had been held in the period of 2018–2019, before new WHO recommendations on Mtb DST (including recommendations of the use of critical concentrations for RIF as 0,5 ml/l and exclusion of Kn and Cap from the list of antituberculosis drugs to be tested) and new definitions were issued [18, 19].

Molecular-genetic analysis of ClIs was performed with commercially available microarray test-systems (TB-TEST,BIOCHIP-IMB, Russia) which were developed by the Institute of molecular biology named after V.A. Engelhard of the Russian Academy of Science. Assay procedures include decontamination of biological specimens, DNA extraction, two consecutive multiplex PCR and PCR-products (amplicons) hybridization on a biologic microchip [47, 48]. Detection of hybridization results, registration, and interpretation were performed with Chipdetector portable fluorescence analyzer. ClIs belonging to Mtb complex were identified by repetitive DNA IS6110, no species identification within the complex was possible, although members of the complex other than *M. tuberculosis* are extremely rare in Russia.

### DNA extraction

Mbt DNA was extracted from Mtb culture using TB-TEST extraction system (BIOCHIP IMB Ltd., Moscow, Russian Federation) following the manufacturer’s protocol.

Genotyping was carried out by microarray technology using TB-TEST (IMB-BIOCHIP, Russia). This method allowed to identify Mtb genotypes endemic for the Russian Federation (Beijing, Beijing B0/W148, LAM, Haarlem, Ural and others) by detection of genotype-specific single nucleotide polymorphism according the algorithm as in [7, 49].

Spoligotyping (Spolygochip, IMB-BIOCHIP, Russia) was used consequently for subtyping of non-Beijing isolates. The resulting record of 43 symbols of binary code per sample was obtained by using the “ImaGeWare” software package and compared to SITVIT2 database (http://www.pasteur-guadeloupe.fr:8081/SITVIT2/index.jsp) Newly reported lineages [50, 51] were not identified.

Genotyping tests were performed according to the manufacturer’s instructions.

Detection of mutations conferring DR was carried out by microarray TB-TEST. The test detects mutations most frequently reported to confer DR in Mtb. Substitutional mutations are presented by substituted amino acids: Arg – Arginine, Asn – Asparagine, Asp-Aspartic acid, Cys – Cysteine, Gln – Glutamine, Glu- Glutamic acid, Gly – Glycine, Ile – Isoleucine, Leu – Leucine, Lys – Lysine, Met-Methionine, Phe – Phenylalanine, Pro – Proline, Ser –Serine, Thr- Threonine, Trp – Tryptophan, Tyr – Tyrosine, Val – Valine. Mutations in regulatory regions of inhA, eis, rrs genes were designated by one-letter code: a – adenine, T- thymine, g- guanine, c - cytosine. Deletions are indicated as Del. Mutations in regulatory region of *inhA eis, rrs* genes are designated by one-letter code: a – adenine, t- thymine, g – guanine, c- cytosine.

All loci and mutations detectable by the test-system used are listed in columns “Loci and types of mutations” of the Tables 3, 4, 5, 6 and 7.

### Statistical analysis

95% confidence intervals (CI 95%) were calculated for prevalence rates (PR) – the rate (%) of genotypes, mutation loci and types. Fisher’s exact test in analysis of two by two tables were used to assess differences between groups of data. Accepted Fisher’s statistical significance level *p* was 0,05. Statistical analysis was done with Statistica software version 12.

## Data Availability

Data supporting the results reported in the article (genotyping and mutations) are presented in the database of the Ural Research Institute of Phthisiopulmonology -Branch of NMRC PhID, Ekaterinburg, Russian Federation: “Database of *Mycobacterium tuberculosis* clinical isolates’ genotypes and mutations, conferring resistance to Isoniazid (INH), Rifampicin (RIF), aminoglycosides (AG) and fluoroquinolones (FQ)” https://urniif.ru/science/docs/genomes/
